# Comparison of the effect on bone healing process of different implants used in minimally invasive plate osteosynthesis: limited contact dynamic compression plate versus locking compression plate

**DOI:** 10.1038/srep37902

**Published:** 2016-11-25

**Authors:** Zichao Xue, Haitao Xu, Haoliang Ding, Hui Qin, Zhiquan An

**Affiliations:** 1 Department of Orthopedic Surgery, Shanghai Jiao Tong University Affiliated Sixth People’s Hospital, No. 600 Yishan Road, Shanghai, 200233, China

## Abstract

Minimally invasive plate osteosynthesis (MIPO) has been widely accepted because of its satisfactory clinical outcomes. However, the implant construct that works best for MIPO remains controversial. Different plate designs result in different influence mechanisms to blood flow. In this study, we created ulnar fractures in 42 beagle dogs and fixed the fractures using MIPO. The dogs were randomly divided into two groups and were fixed with a limited contact dynamic compression plate (LC-DCP) or a locking compression plate (LCP). Our study showed that with MIPO, there was no significant difference between the LCP and the LC-DCP in terms of fracture fixation, bone formation, or mineralization. Combined with the previous literature, we inferred that the healing process is affected by the quality of fracture reduction more than plate selection.

Minimally invasive plate osteosynthesis (MIPO) has gained popularity because it minimizes dissection of broken bone fragments and therefore preserves vascularity and increases the healing potential^1^,^2^,^3^,^4^,^5^,^6^,^7^,^8^,^9^,^10^,^11^,^12^. It provides adequate stability and is a safe, effective method for both comminuted and simple fractures^6^,^11^,^13^,^14^. However, the type of plate fixation that works best for MIPO is not clear. The conventional plate was originally used with the MIPO technique^6^,^9^,^10^,^11^,^14^. The first was the dynamic compression plate (DCP). However, the DCP was superseded with the limited contact dynamic compression plate (LC-DCP) for its shortcoming of extensive contact of the undersurface that interferes with the periosteal blood flow^15^,^16^. Later, the locking compression plate (LCP) was increasingly used for its theoretical advantages^12^,^17^,^18^,^19^,^20^,^21^,^22^,^23^,^24^,^25^,^26^,^27^.

Although different designs of these two plates result in different influence mechanisms to blood flow, comparison studies of the conventional plate and LCP that addressed their clinical use and their biomechanics showed no significant difference between the two types of plates^20^,^28^,^29^,^30^,^31^. To the best of our knowledge, no study has reported analyzed differences in the healing process when using two types of plates with the MIPO technique. Therefore, we conducted this study to compare the healing process of fractures treated with LC-DCP or LCP by the MIPO technique in terms of callus formation and bone mineralization.

## Results

### General and radiographic observations

All operations were completed with no intraoperative complications. There was no significant difference in operation time between the two groups (*p* = 0.15) (Table 1). All dogs recovered well postoperatively with no signs of any complications.

**Figure 1:**
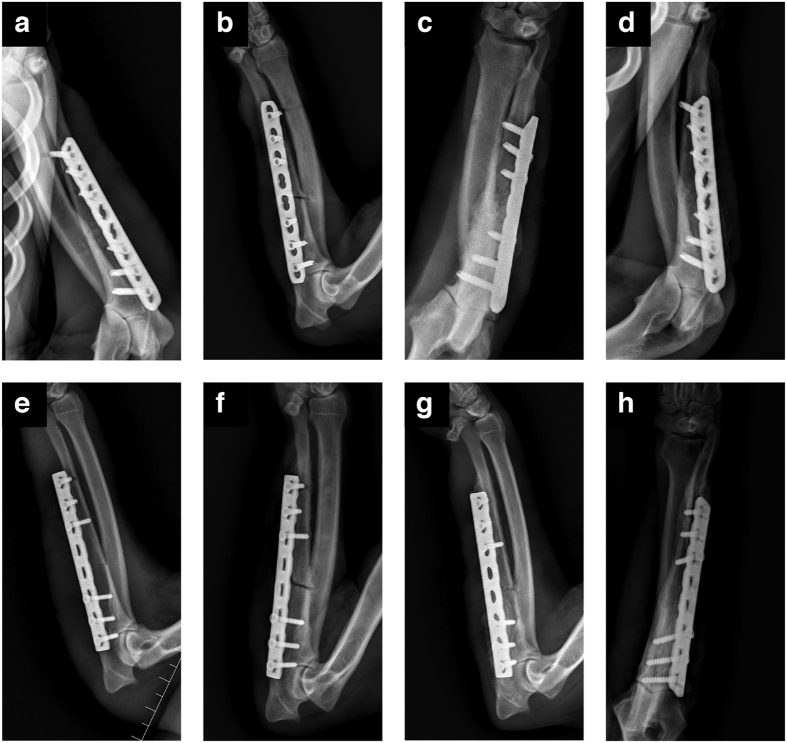
Radiographic images of fractures fixed with plates on day 1 and 4, 8, and 12 weeks after the operation. (**a–d**) Locking compression plate (LCP). (**e–h**) Dynamic compression plate (LC-DCP).

Using the radiographs obtained right after the operation, one author determined the interfragmentary gap, mediolateral translation, and angulation. There was no significant difference in reduction between the two groups (Table 1). Four weeks after the operation, it was observed in most dogs that some callus had formed at the fracture site, and the fracture line was still visible. Four dogs from the LCP group and three dogs from the LC-DCP group had achieved fracture union with complete callus. At 8 weeks, the results showed that the excess callus was reabsorbed, and the callus mass volume decreased. Of the 14 dogs in the LCP group, 12 achieved fracture union, as did 13 of the 14 dogs in the LC-DCP group. All dogs in both groups showed radiographic fracture union by 12 weeks post operation (Fig. 1).

### Evaluation of Micro-CT

**Figure 2:**
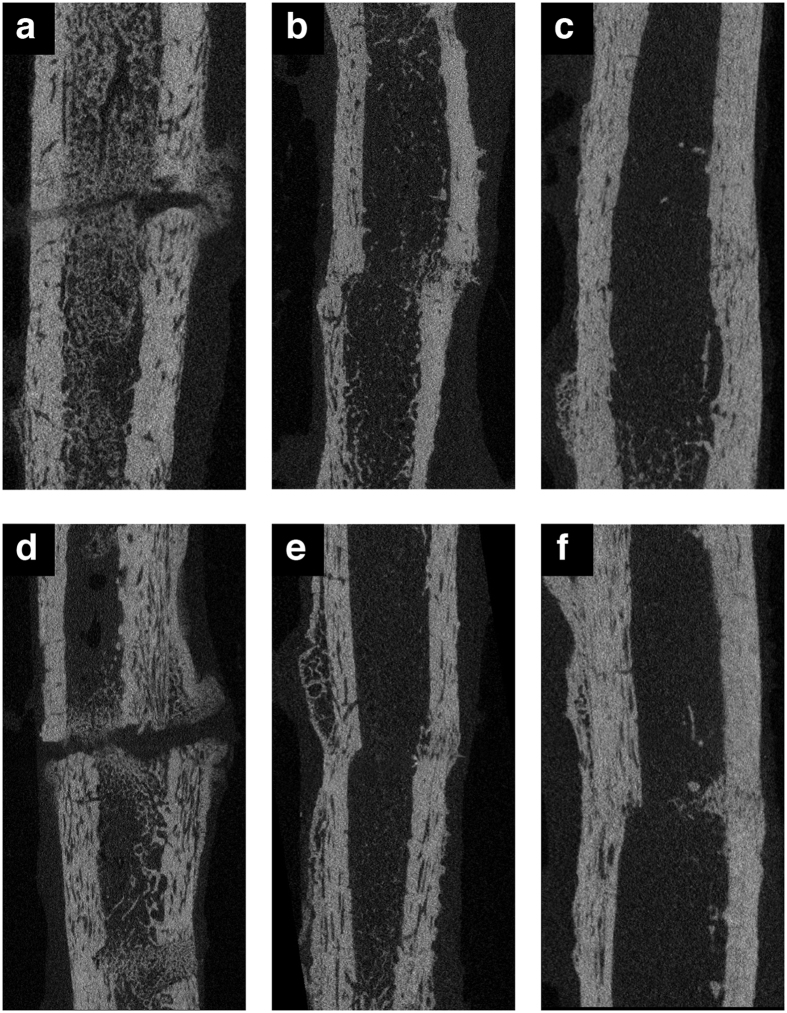
Micro-CT images of fractures at 4, 8, and 12 weeks after the operation. (**a–c**) LCP group. (**d–f**) LC-DCP group.

The mineralization of callus and bone was examined by micro-CT. The images showed findings similar to those on plain radiographs (Fig. 2). In both groups, a callus had formed around the fracture site during the first 4 weeks post operation. The callus, formed mainly from the periosteum, was observed in the fracture gap, and the fracture line was still visible. During the next 4 weeks, the amount of callus was reduced. A mineralized callus bridged the fracture gap, and endosteal callus formation was active. The fracture line had become vague on the images. During the last 4 weeks, the fracture was completely healed, and the bone was remodeled.

The volume and relative density of the callus and newly formed bone were calculated and documented (Table 2). In both groups, the volumes of callus and bone decreased gradually at various time points. The difference in the volume of callus or bone between the two groups at each time point was not significant. The volume ratio of the callus and bone also declined with increasing time. There was no significant difference in the volume ratios of the two groups. The relative density of callus and bone stayed almost the same during the whole healing process. There was no significant difference between the two groups regarding the relative density of callus or bone.

### Fluorochrome labeling histomophometrical analysis

**Figure 3:**
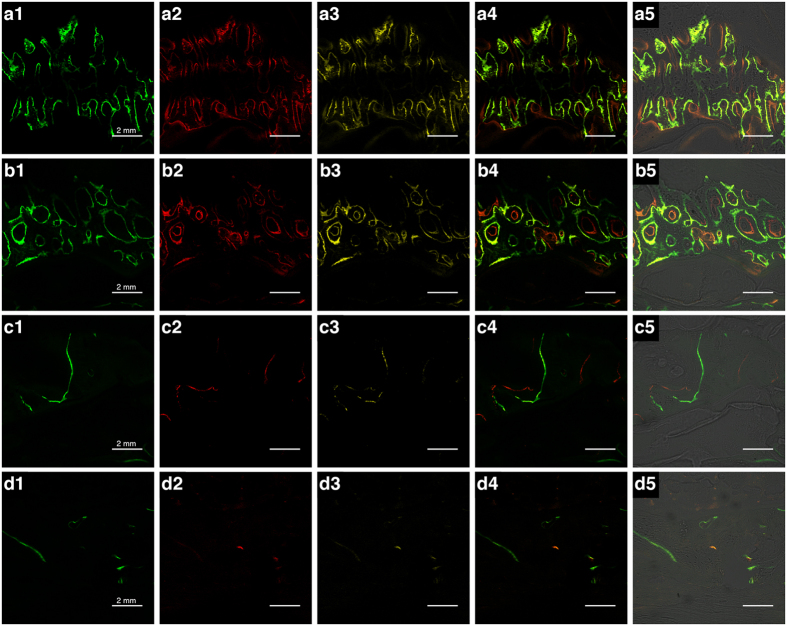
Sequential fluorescence labeling images of CA (green), AL (red), and TE (yellow). The images (**a1,b1**), (**a2,b2**), (**a3,b3**), (**c1,d1**), (**c2,d2**), and (**c3,d3**) represent the labeling on the same day as the operation and at 2, 4, 6, 8, and 10 weeks post operation. (**a4,b4,c4 and d4**) represent the merged images of the three fluorochromes for the same group. (**a5,b5,c5 and d5**) represent the merged images of the three fluorochromes using plain confocal laser microscopy (scale bar = 2 mm). A and C represent the LCP group. B and D represent the LC-DCP group.

**Figure 4:**
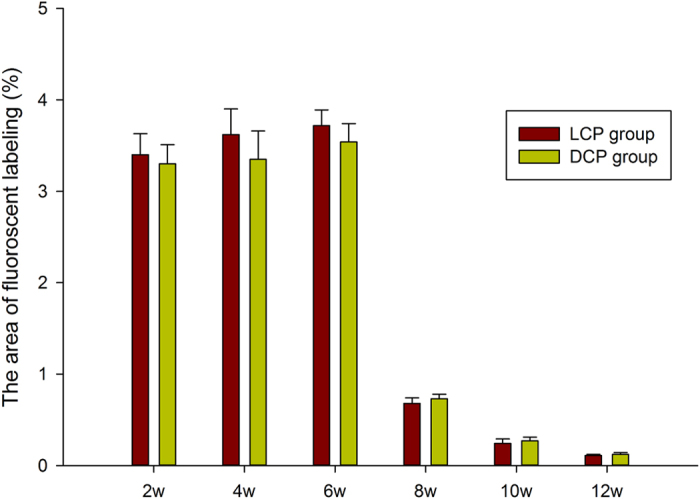
Percentages of the fluorochrome areas on fluorescence labeling images.

Bone formation and mineralization were evaluated histomorphometrically by calcein (CA), alizarin (AL) and tetracycline (TE) fluorescent quantification. The percentage of fluorochrome labeling represented the level of callus mineralization. During the first 2 weeks after surgery, the percentage of CA labeling was 3.40 ± 0.23% in the LCP group and 3.30 ± 0.21% in the LC-DCP group (*p* = 0.41) (Fig. 3). During the next 2 weeks, the percentage of AL labeling was 3.62 ± 0.28% in the LCP group and 3.35 ± 0.31% in the LC-DCP group (*p* = 0.11) (Fig. 3). During the period 4–6 weeks post operation, the percentage of TE labeling was 3.72 ± 0.17% in the LCP group and 3.54 ± 0.20% in the LC-DCP group (*p* = 0.09) (Fig. 3). New bone formation and mineralization were active in both groups during the first 6 weeks after surgery (Fig. 4). Between 6 and 8 weeks post operation, the percentage of labeling decreased to 0.68 ± 0.06% in the LCP group and to 0.73 ± 0.05% in the LC-DCP group (Fig. 3). These decreases may indicate the occurrence of callus reabsorption and bone remodeling. After 8 weeks, the percentage of labeling decreased to a low level (Fig. 3). No significant differences were detected between the two groups with regard to labeling during any time period (Fig. 4).

### Histological findings

**Figure 5:**
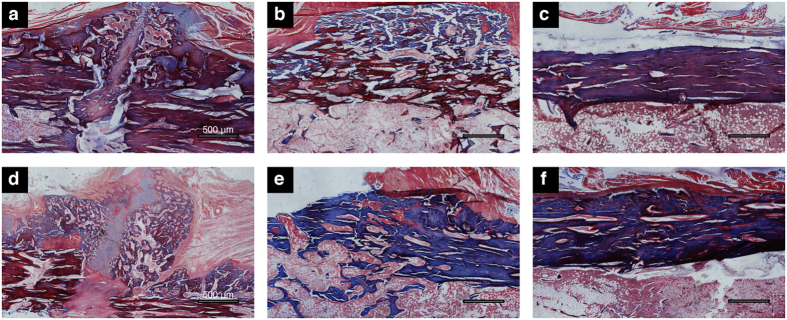
Masson-stained histological sections. The whole images are representative slices of the two groups. LCP group at 4 weeks (**a**), 8 weeks (**b**), and 12 weeks (**c**). LC-DCP group at 4 weeks (**d**), 8 weeks (**e**), and 12 weeks (**f**). Scale bar = 500 μm.

Histological observation was performed under light microscopy. Generally, the findings in the two groups were similar. Four weeks after surgery, 6 of the 7 dogs from the LCP group and all 7 dogs from the LC-DCP group showed callus formation at the fracture site. The fracture line could be clearly identified in the field (Fig. 5). The other one from the LCP group showed fracture union with callus. By the end of 8 weeks, the fracture gap was bridged with new bone, and the gap had disappeared in all 7 dogs from each group. The callus mass stopped growing and became smaller (Fig. 5). At 12 weeks, the callus had been completely resorbed, and the newly formed bone had been remodeled in all 14 dogs from both groups. The fracture site had the same appearance as that of the undisturbed bone nearby (Fig. 5).

## Discussion

The MIPO technique has gained popularity recently because of its satisfactory clinical outcomes^1^,^2^,^3^,^4^,^5^,^6^,^7^,^8^,^9^,^10^,^11^,^12^. This method minimizes disruption of soft tissue and preserves the blood supply to the fracture site. However, the optimum plate used for fixation with the MIPO technique remains unclear. When MIPO was introduced, conventional plating with DCP was used^6^,^9^,^10^,^11^,^14^. Later, the DCP was superseded with LC-DCP for its flat undersurface^15^,^32^. LCP, which developed later than DCP and LC-DCP, acts as an internal fixator. Theoretically, one of its advantages over a conventional compression plate is that it causes less damage to the bone’s blood supply^12^,^22^,^23^,^24^,^25^,^26^,^27^. Therefore, it is supposed to be an optimal implant for MIPO and has been widely used with the MIPO technique^33^,^34^,^35^,^36^.

Comparative analysis, however, revealed no differences between these two implants clinically or biomechanically^20^,^28^,^31^. To our knowledge, no study has compared the mechanism of the healing process of these two implants using MIPO. To provide information from this perspective, we performed this study to compare the formation and mineralization of callus and bone in an *in vivo* experiment. No significant differences were found between the two implants for any of the parameters measured.

With a conventional plate, the fixation stability results from the friction between the plate and bone. To obtain this, a larger perpendicular force has to be applied to press the plate to the bone; this increased force may disturb the periosteal perfusion^30^,^32^,^37^. The LCP, however, does not rely on the friction between the plate and bone but depends on the angular stable construct because of the locking head screws^27^. Because the screws are locked in the plate, the forces are transferred from the bone to the fixator across the screw-plate threaded connection. This construct maintains a potential space between the plate and the bone, requiring no compression to achieve stability, thus minimizing the damage to the periosteum^27^,^37^. Although this advantage is supposed to lead to rapid bone healing, it remains a theory^30^,^38^. The present study showed that despite the different concepts of fixation, there was no difference between the LC-DCP and LCP in terms of callus formation and mineralization when treating fractures with the MIPO technique.

The LCP works as an internal fixator and is of particular advantage in an MIPO^27^. The LC-DCP, however, can also be considered to be an internal fixator^32^. It reduces the bone-plate contact by 50% to minimize the disruption to periosteal blood flow^32^,^38^. Multiple studies have shown that LC-DCP could protect the blood supply and prevent osteoporosis in both the short and long term^15^,^16^,^32^,^39^. The undercuts of LC-DCP allows a small amount of callus formation. This callus, although small, increases the strength at a very critical location^32^. Therefore, LC-DCP supports the MIPO like LCP does. Callus formation that may result in a solid union depends on a good blood supply. As proved by our previous *in vivo* study, MIPO could promote early callus formation and mineralization^40^. Under this circumstance, we infer that these two plates may have equal effects in preserving the blood supply. Further studies are needed regarding this aspect.

Ashutosh concluded that the fracture healing pattern was determined more by the fixation principle than by the selection of plates^41^. With the MIPO technique, the plates function as bridging plates^42^,^43^. Experience has shown that this pattern is associated with a high risk of failure with regard to simple fractures^34^,^44^. However, extensive studies have shown that the MIPO technique used with a bridging plate can provide adequate stability even for simple fractures^6^,^11^,^13^,^14^. In our study, all simple fractures healed with some but not much callus. Because the bridging structure can provide enough stability in the MIPO technique, it is likely that the healing pattern is related to fracture reduction.

This is consistent with previous studies that found that the healing pattern was affected by the quality of reduction rather than by the type of implant^30^,^38^. These studies found that the healing pattern was affected by the quality of reduction rather than by the type of implant. When anatomical reduction was achieved, there was minimal callus formation. In Bruno’s study, patients treated by MIPO and a broad DCP healed with no evidence of callus^11^.

Because the fracture is usually reduced by indirect reduction when the MIPO technique is used, it is technically demanding to achieve anatomical reduction. The LCP may have an advantage in this regard. The LCP does not need to be precontoured, and this suppresses the risk of primary loss of reduction. However, in the present study, there was no significant difference in either the reduction quality or surgery time.

In conclusion, the present study showed that with MIPO, there was no significant difference between the LCP and LC-DCP in terms of fracture fixation, callus formation or mineralization. This may indicate that both plates have an equal effect on preserving blood supply. Combined with the previous literature, we inferred that the healing process is affected by the quality of fracture reduction more than plate selection.

## Methods

### Ethics statements

The Animal Care and Use Committee of Shanghai Sixth People’s Hospital affiliated with Shanghai Jiao Tong University approved this study. All animal experiments including surgical methods were performed in accordance with the approved guidelines by the Animal Experimental Center of Shanghai Sixth People’s Hospital affiliated with Shanghai Jiao Tong University.

### Animal conditions and grouping

We were supplied with 42 male beagle dogs (2 years old, average weight 16 kg) from the Agriculture College of Shanghai Jiao Tong University. The dogs were randomly divided into two groups and were kept separately with sufficient water and a standard dry food diet (Laboratory Canine Diet, Animal Experimental Center of Shanghai Sixth People’s Hospital) ad libitum. The housing facility is in accordance with national standard Laboratory Animal Requirements of Environment and Housing Facilities (GB 14925-2010). Dogs were housed in kennels of 2.25 m^2^ and played in the activity room for one hour in the morning and afternoon.

### Animal models

Animal models were created according to the protocol previously described^40^. Under general anesthesia, a small incision was made at the middle of the lateral side of the dog’s forearm. An osteotomy was performed using an oscillating saw to create a transverse fracture on the ulna. Next, two small incisions were made proximal and distal to the fracture site. A submuscular extraperiosteal tunnel was created with blunt dissection. We then inserted the plate (3.5 mm system, 8-hole LCP or LC-DCP in regard to different groups) through the tunnel and fixed it with three screws (locking screws for LCP and cortical screws for LC-DCP) at each end.

Postoperatively, analgesic (lidocaine 2 mg/kg) was given once within 24 hours and antibiotic medication (ampicillin sodium 20 mg/kg) was administered for 5 days. We randomly selected seven dogs in each group and euthanized them with 10% potassium chloride at 4, 8, and 12 weeks post operation. The samples, consisting of the entire ulna and radius, were collected and kept in buffered formalin (10%, pH 7.4) for later testing.

### Sequential fluorescent labeling

Fluorescence labeling was carried out postoperatively to observe bone mineralization and deposition at various time periods. For the dogs euthanized at 8 weeks, calcein 20 mg/kg (CA), alizarin red 30 mg/kg (AL), and tetracycline (TE) 25 mg/kg (all obtained from Sigma-Aldrich, St. Louis, MO, USA) were injected subcutaneously right after the operation and at 2 and 4 weeks after the operation, respectively. For the dogs euthanized at 12 weeks, the injections were at 6, 8, and 10 weeks after the operation, respectively.

### Radiographic observation

Radiographic images were obtained 1 day and 4, 8, and 12 weeks post operation with the dogs under general anesthesia. The bulb tube parameters were 50–55 kV and 5–10 mAs. The fracture union was defined as the presence of a bridged callus in at least three of four cortices on two radiographic views^45^.

### Evaluation of micro-CT

For evaluation of the bone and mineralized callus, all samples were scanned using micro-computed tomography (micro-CT) (SkyScan 1176 compact X-ray MicroCT scanner; Bruker, Brussels, Belgium) with the beam set at 90 kV and 270 μA and reconstructed at 18 μm isotropic resolution (NRecon Program; Bruker).

As in previous publications, the mineralized tissues were classified as bone or callus depending on its density relative to that of the undisturbed cortical bone^45^. A callus was defined as bone having a density of 35–70% of the maximum density of the undisturbed cortical bone. Bone was defined as having a density of >70% of the maximum density. A 10-mm length of a cylindrical volume of interest was selected for analyzing the fracture site. It was centered at the midpoint of the fracture in the longitudinal view.

Mineralized callus volume and density and bone volume and density were determined and analyzed to compare the differences in mineralization of the two groups. SkyScan Dataviewer and SkyScan CTan (Bruker) were used for these evaluations.

### Sample preparation and histomorphometric observation

The samples were dehydrated in alcohol of ascending concentrations, from 70% to 100%, and then embedded in polymethylmethacrylate. Then, the samples were sliced into 8-μm thick sections with a microtome (Leica, Hamburg, Germany).

Three sections were randomly chosen from each sample for fluorescence labeling analysis. The observation area was centered at the fracture site. This step was conducted using a confocal laser scanning microscope (LSM710; Carl Zeiss, Oberkochen, Germany) with the excitation/emission wavelength of chelating fluorochrome settings as follows: 488/517 nm (calcein: green), 543/617 nm (alizarin red: red), and 405/615 nm (tetracycline: yellow). Five photographs were taken (10×) for each section from the same visual field: three fluorescence microscopy images of each fluorochrome (calcein, alizarin red, and tetracycline); one merged image of all three fluorescent labels; and one merged image of the transmission light.

The microscopy images were digitally saved and evaluated histomorphometrically using a picture-analysis software system (Image-Pro Plus; Media Cybernetics, Rockville, MD, USA). The software calculated the percentage of the total mineralization area by counting the number of pixels labeled for each fluorochrome on each image. The results reflected the bone formation and mineralization at different time periods. The sections were then further treated with Masson staining for histological observation of the formation and mineralization of the callus.

### Statistical analysis

The results are presented as the mean ± standard deviation. Differences between the two groups were determined by *t*-tests. A value of *p* ≤ 0.05 was considered to indicate significance. All statistical analyses were performed using the SAS 8.02 statistical software package (SAS Institute, Cary, NC, USA).

## Additional Information

**How to cite this article**: Xue, Z. *et al.* Comparison of different implants used in minimally invasive plate osteosynthesis: limited contact dynamic compression plate versus locking compression plate. *Sci. Rep.* **6**, 37902; doi: 10.1038/srep37902 (2016).

**Publisher’s note:** Springer Nature remains neutral with regard to jurisdictional claims in published maps and institutional affiliations.

## Figures and Tables

**Table 1:** Surgery and reduction data by minimally invasive plate osteosynthesis.

	LCP Group	LC-DCP Group	*p*-value
Time of surgery (m)	33.67 ± 5.96	36.29 ± 5.71	0.15
Interfragmentary gap (mm)	0.31 ± 0.10	0.28 ± 0.10	0.27
Mediolateral translation (mm)	0.78 ± 0.48	0.81 ± 0.53	0.83
Varus-valgus angulation (degree)	3.04 ± 1.91	2.90 ± 1.89	0.81

**Table 2:** Data analyses of Micro-CT scan results.

	LCP Group	LC-DCP Group	*p*-value	
4 weeks				
Mineralized callus volume (mm^3^)	306.86 ± 26.94	283.09 ± 30.76	0.15	
Relative mineralized callus density	0.156 ± 0.008	0.152 ± 0.006	0.28	
Bone volume (mm^3^)	388.57 ± 14.99	372.15 ± 18.11	0.10	
Relative bone mineral density	0.183 ± 0.009	0.183 ± 0.008	0.85	
Callus volume/Bone volume	0.79 ± 0.09	0.76 ± 0.11	0.60	
8 weeks				
Mineralized callus volume (mm^3^)	213.14 ± 16.05	230.86 ± 20.05	0.09	
Relative mineralized callus density	0.154 ± 0.008	0.154 ± 0.006	0.83	
Bone volume (mm^3^)	473.42 ± 33.90	504.67 ± 36.17	0.12	
Relative bone mineral density	0.182 ± 0.008	0.184 ± 0.008	0.61	
Callus volume/Bone volume	0.45 ± 0.04	0.46 ± 0.05	0.75	
12 weeks				
Mineralized callus volume (mm^3^)	140.39 ± 10.23	144.86 ± 12.23	0.45	
Relative mineralized callus density	0.155 ± 0.007	0.157 ± 0.005	0.46	
Bone volume (mm^3^)	494.52 ± 32.56	489.14 ± 36.69	0.80	
Relative bone mineral density	0.182 ± 0.007	0.180 ± 0.008	0.68	
Callus volume/Bone volume	0.28 ± 0.02	0.30 ± 0.02	0.35	
